# Naringin in Ganshuang Granule suppresses activation of hepatic stellate cells for anti‐fibrosis effect by inhibition of mammalian target of rapamycin

**DOI:** 10.1111/jcmm.12994

**Published:** 2016-09-30

**Authors:** Hongbo Shi, Honglin Shi, Feng Ren, Dexi Chen, Yu Chen, Zhongping Duan

**Affiliations:** ^1^ Beijing Youan HospitalCapital Medical UniversityBeijingChina; ^2^ Beijing Institute of HepatologyBeijingChina

**Keywords:** naringin, hepatic stellate cells, autophagy, mammalian target of rapamycin

## Abstract

A previous study has demonstrated that Ganshuang granule (GSG) plays an anti‐fibrotic role partially by deactivation of hepatic stellate cells (HSCs). In HSCs activation, mammalian target of rapamycin (mTOR)‐autophagy plays an important role. We attempted to investigate the role of mTOR‐autophagy in anti‐fibrotic effect of GSG. The cirrhotic mouse model was prepared to demonstrate the anti‐fibrosis effect of GSG. High performance liquid chromatography (HPLC) analyses were used to identify the active component of GSG. The primary mouse HSCs were isolated and naringin was added into activated HSCs to observe its anti‐fibrotic effect. 3‐methyladenine (3‐MA) and Insulin‐like growth factor‐1 (IGF‐1) was added, respectively, into fully activated HSCs to explore the role of autophagy and mTOR. GSG played an anti‐fibrotic role through deactivation of HSCs in cirrhotic mouse model. The concentration of naringin was highest in GSG by HPLC analyses and naringin markedly suppressed HSCs activation *in vitro*, which suggested that naringin was the main active component of GSG. The deactivation of HSCs caused by naringin was not because of the autophagic activation but mTOR inhibition, which was supported by the following evidence: first, naringin induced autophagic activation, but when autophagy was blocked by 3‐MA, deactivation of HSCs was not attenuated or reversed. Second, naringin inhibited mTOR pathway, meanwhile when mTOR was activated by IGF‐1, deactivation of HSCs was reversed. In conclusion, we have demonstrated naringin in GSG suppressed activation of HSCs for anti‐fibrosis effect by inhibition of mTOR, indicating a potential therapeutic application for liver cirrhosis.

## Introduction

Hepatic fibrosis can develop into cirrhosis within 1–10 years with a 7‐ to 10‐year liver‐related mortality of 12–25% 1. Unfortunately, effective clinical therapies are still lacking 2. Some Chinese medicine herbs or prescriptions may offer hope in treating cirrhosis 3, 4. Ganshuang granule (GSG), based on prescription of a traditional Chinese medicine, has been used for treating various chronic liver diseases. It is believed by the theory of traditional Chinese medicine that GSG functions through protecting the liver, strengthening the spleen, clearing heat and stasis and resolving hard lump. However, the mechanism through which GSG protects the liver still remains unknown.

A previous study has demonstrated that GSG plays an anti‐fibrotic role partially by suppressing the activation of hepatic stellate cells (HSCs) 5, 6. In HSCs activation, mammalian target of rapamycin (mTOR)‐autophagy pathway plays an important role. Jin *et al*. found that activation of autophagy causes activation of rat HSCs through calcium‐dependent AMPK/mTOR and PKCθ pathway under hypoxic stress 7. So we speculated that mTOR‐autophagy pathway may act as an important role in anti‐fibrosis effect of GSG.

The present study discovered that the herbal prescription GSG which has been known for its protective effects on the liver reverses activated HSCs to their quiescent phenotype in mouse model. High performance liquid chromatography (HPLC) analyses identify naringin as the main active component of GSG. Naringin achieves the anti‐fibrotic effect by suppressing activation of HSCs through mTOR pathway.

## Materials and methods

### Animal experiments

Male Balb/c mice (6 weeks of age; weight, 25–30 g) were provided by the Animal Center at the Academy of Military Medical Sciences. All animals were placed in a pathogen‐free environment and maintained in 12‐h dark/light cycles at 22–24°C and 30–40% humidity. 30 mice were randomly divided into the model group (*n* = 20) and the control group (*n* = 10). The random method was application of random number table. The model group was intraperitoneally injected with a mixture of CCl_4_ (40%) and oil (60%) at a dose of 2 ml/kg body weight twice a week for 12 weeks. The control group was injected with the same volume of oil at the same time and position as the model group. At week 12, CCl_4_ injection was stopped and the mice were subjected to additional treatment for 2 weeks: (i) Control group: mice in control group were fed with normal diet; (ii) Model group: mice in model group were treated intragastrically with distilled water equal to the volume of GSG besides normal diet; (iii) GSG group: mice in model group were treated intragastrically with GSG at a dose of 4 g/(kg·day) besides normal diet.

### HPLC of GSG

GSG was developed by the Buchang Pharmacy Limited Company in Xi'an province of China. The standard product such as paeoniflorin, polydatin, ferulic acid, naringin, neohesperidin, saikosaponin a, saikosaponin d, tanshinone IIA and emodin were purchased from National Institutes for Food and Drug Control of China (NIFDCC, Beijing). The chemical pattern was analysed using high performance liquid chromatography (HPLC, Waters 2695, Milford, MA, USA) with UV detection at 210 nm. The analysis was performed with a CAPCELL PAK C18 MG column (250 mm × 4.6 mm × 5 μm, Shiseido, Japan) at 40°C. The compounds were eluted (elution buffer A, water; elution buffer B, acetonitrile) at a flow rate of 1 ml/min. using a gradient program.

### Isolation and treatment of primary mouse HSCs

The mouse livers were perfused with Hank's solution containing collagenase, and viable HSCs were isolated by percoll isodensity centrifugation as described previously 8. HSCs were observed in UV light of microscope at day 1, 3, 7 after isolation. Naringin (20 ng/ml, NIFDCC, China) was added into day 1 HSCs for 24 hrs and day 3 activating or day 7 fully activated HSCs for 48 hrs. 3‐methyladenine (3‐MA, 750 ng/ml, Sigma‐Aldrich, St. Louis, MO, USA) was added into day 7 fully activated HSCs for 2 hrs. Insulin‐like growth factor‐1 (IGF‐1, 500 ng/ml, Sigma‐Aldrich) was added into day 7 fully activated HSCs for 2 hrs.

### Masson trichrome staining

The samples were deparaffinized and rehydrated with 100% alcohol, 95% alcohol and 70% alcohol. The samples were then washed in distilled water and rinsed in running tap water for 5–10 min. before staining in Biebrich scarlet‐acid fuchsin solution for 10–15 min., washed in distilled water and differentiated in phosphomolybdic‐phosphotungstic acid solution for 10–15 min. or until the collagen was not red. The sections were transferred directly (without rinsing) to an aniline blue solution and stained for 5–10 min. before being rinsed briefly in distilled water and differentiated in 1% acetic acid solution for 2–5 min. Then, the samples were washed in distilled water and dehydrated quickly with 95% ethyl alcohol, absolute ethyl alcohol and clear in xylene. The samples were then mounted with resinous mounting medium.

### Immunofluorescence

The sections were blocked with a blocking buffer (5% goat serum and 0.2% bovine serum albumin at room temperature for 30 min. and then washed three times with phosphate buffered saline (PBS). The primary antibody anti‐smooth muscle actin (anti‐SMA) and anti‐desmin (Abbiotec, San Diego, CA, USA, 1: 400) was applied at room temperature for 1 hr. Then, the samples were washed, and the secondary antibody [goat anti‐mouse and goat anti‐rabbit F(ab’)2 fragment of IgG (H+L) antibody, Invitrogen, Carlsbad, CA, USA, 1:400] was applied at room temperature for 30 min. The sections were counterstained with DAPI. All images were obtained using an inverted fluorescence microscope (Nikon Eclipse E800, Tokyo, Japan).

### Western blotting analysis

After the designated treatments, Liver tissues and cell pellets were lysed with RIPA buffer supplemented with protease inhibitors. Total proteins (50 μg) were separated *via* 12% SDS‐polyacrylamide gel electrophoresis (PAGE) and transferred to polyvinylidene difluoride (PVDF) membranes. The membranes were incubated overnight with rabbit antibodies against LC3B (Sigma‐Aldrich), p62, Atg5, Beclin‐1and β‐actin (Cell signaling, CA, USA) at 4°C. Then, the membranes were treated with a horseradish peroxidase‐conjugated goat anti‐rabbit secondary antibody (Cell signaling, Danvers, MA, USA) and developed with a chemiluminescent substrate (Thermo Fisher Scientific, Rockford, IL, USA). Densitometry analysis was performed using ImageJ software (National Institute of Health, Bethesda, MD, USA), and the relative levels of protein in each group were normalized to the loading control.

### Quantitative polymerase chain reaction

Total RNA was extracted using the Trizol kit (Invitrogen, USA), according to the manufacturer's instructions. The first strand cDNA was synthesized from 5 μg of RNA (Superscript III cDNA Synthesis Kit, Invitrogen), and the mRNA levels of collagen‐I, SMA and glyceraldehyde 3‐phosphate dehydrogenase (GADPH) were determined by quantitative polymerase chain reaction qPCR using the SYBR Green PCR Kit (Invitrogen) in a real‐time PCR system (ABI PRISM7300, Foster, CA, USA). The relative quantification cycle threshold (CT) value for each primer was normalized to the internal primer.

### Statistical analyses

One‐way analysis of variance (anova), followed by a post hoc LSD test, was used to compare the differences between multiple groups. Independent‐sample *t*‐test was used to compare the differences between two groups. *P* < 0.05 was considered significant. All data were analysed with SPSS software, IBM SPSS, Armonk, NY, USA, version 11.5.

## Results

### GSG depresses liver fibrosis and activation of HSCs in mouse model

Compared with model group, the mice treated with GSG had fewer fibrous connective tissues and pseudolobules in Masson's staining. These results showed that the level of fibrosis in model group was higher than that in GSG group, which suggested that GSG played an anti‐fibrotic role in the cirrhotic mouse model. Compare with model group, the expression of SMA was decreased, and there was less nuclear deformation and disordered structure in the liver tissues of the mice in GSG group. In addition, the difference in the mRNA expression of collagen‐I and SMA among groups was significant (collagen‐I: *F* = 31.254, *P* = 0.000; SMA: *F* = 6.204, *P* = 0.008), and the expression of collagen‐I and SMA in model group was higher than those in GSG group. These results demonstrated the anti‐fibrotic effect of GSG in terms of the suppression of the activation of HSCs.

### Identification of active component of GSG

As showed in the HPLC fingerprint, the different components were eluted at different time‐points. The standard curves between concentration and peak area were drawn and concentration of different components in GSG was calculated by peak area. Nine active components in GSG were identified such as paeoniflorin (peak 1, 2.224 mg/g), polydatin (peak 2, 1.557 mg/g), ferulic acid (peak 3, 0.733 mg/g), naringin (peak 4, 6.128 mg/g), neohesperidin (peak 5, 2.783 mg/g), saikosaponin a (peak 6, 0.039 mg/g), saikosaponin d (peak 7, 0.469 mg/g), tanshinone IIA (peak 8, 0.484 mg/g) and emodin (peak 9, 0.102 mg/g). The concentration of naringin was highest in GSG, which suggested that naringin may be the main active component of GSG.

### Naringin suppresses activation of primary mouse HSCs

To explore the mechanisms of the demonstrated anti‐fibrotic effect of GSG at the cellular level, primary mouse HSCs were treated with naringin or the solvent as a control. Mouse HSCs cultured on plastic dish spontaneously undergo myofibroblastic transdifferentiation (‘activation’) from day 2 to 3 and become fully activated by day 5 to 7 9. Naringin treatment morphologically reversed activated HSCs to quiescent cells characterized by more lipid drops and less dendrite‐like processes than control in UV light. In addition, naringin markedly suppressed messenger RNA (mRNA) expression of markers for HSC activation such as collagen‐I and SMA. These results supported that naringin was indeed active component that rendered the GSG's effect to inhibit or reverse HSC activation.

### The suppressive effect of naringin on HSCs activation is further enhanced by inhibition of autophagy

To investigate the signalling pathways in suppressive effect of naringin on HSCs activation, autophagy‐related molecules were detected in HSCs treated with naringin. It is generally accepted that autophagy‐related gene (ATG) regulates autophagy, among which ATG6/Beclin1 is a critical regulator at the beginning of nucleation of autophagic vesicles 10. The second involves the conjugation of phosphatidylethanolamine to a LC3 by the sequential action of the Atg4, Atg7 and Atg3. This lipid conjugation leads to the conversion of the soluble form of LC3 (named LC3‐I) to the autophagic vesicle‐associated form (LC3‐II), allowing for the closure of the autophagic vacuole 11. p62, which is a lysosome substrate, is degraded when autophagosomes fuse with lysosomes 12. In our study, the levels of Beclin1 in HSCs treated with naringin were higher than that in control. In primary mouse HSCs, LC3‐II levels were markedly increased by naringin treatment. In addition, the levels of p62 were decreased in HSCs treated with naringin. Collectively, these results indicated that naringin promoted autophagic flux in HSCs.

To further dissect the function of autophagy in naringin suppressing HSCs activation, 3‐MA was added into HSCs to inhibit autophagy. The deactivation of HSCs caused by naringin was further enhanced by the inhibiton of autophagy, which suggested deactivation of HSCs by naringin was not because of the activation of autophagic pathway because blocking of autophagic pathway did not promote HSCs activation. In addition, the mRNA expression of collagen‐I and SMA also revealed a similar trend as morphology. The mRNA expression of collage‐I and SMA was significantly lower in HSCs treated with both naringin and 3‐MA than that in HSCs treated with naringin only. Interestingly, inhibition of autophagy suppressed HSCs activation. Collectively, it suggested that deactivation of HSCs caused by naringin was not a result of the autophagic activation.

### Naringin suppresses activation of HSCs through mTOR pathway

Considering that mTOR pathway is one of the major proliferative pathways, we investigated the role of mTOR in naringin suppressing HSCs activation. mTOR is a serine/threonine kinase, which acts as a central controller in regulating important cellular functions. It exists in two multiprotein complexes: mTOR complex 1(mTORC1) and mTOR complex 2 (mTORC2) 13. The downstream mediators of mTORC1 include p70S6K and S6 14. In our study, naringin treatment showed significant decrease in levels of phosphor‐mTOR (Ser2481, Ser2448), phosphor‐p70S6K (Thr389) and phosphor‐S6 (Ser235/236) in HSCs which suggested mTOR pathways were inhibited by naringin. These findings indicated that the mTOR/p70S6K pathway was likely responsible for suppression of HSCs activation caused by naringin.

To test this hypothesis, we used IGF‐1 to promote the mTOR pathway activation. Remarkably, IGF‐1 treatment promoted HSCs activation, which suggested deactivation of HSCs by naringin was because of the inhibition of mTOR pathway because activation of mTOR pathway reversed the suppressive effect of naringin on HSCs activation. In addition, the mRNA expression of collagen‐I and SMA also revealed a similar trend as morphology. In HSCs treated with naringin, the expression of collagen‐I and SMA was significantly increased in the present of IGF‐I, indicating that the suppressive effect of naringin on HSCs activation was significantly attenuated or completely abolished when mTOR pathway was activated. Therefore, naringin was able to suppress HSCs activation *via* inhibiting mTOR pathway.

## Discussion

HSC activation is considered a major mechanism in the formation of fibrosis and cirrhosis. In the normal adult liver, quiescent HSCs are characterized by desmin expression, lipid drops storage, and extensive dendrite‐like processes. Upon injury, HSCs are activated, lose lipid drops and transform into a myofibroblastic phenotype expressing SMA and excessive extracellular matrix proteins 15. In our study, GSG treatment decreased expression of SMA and collage in the cirrhotic mouse model, which indicated that GSG played an anti‐fibrotic role through suppression of HSCs activation.

Naringin was identified as the main active component of GSG by HPLC analyses. In primary mouse HSCs, naringin treatment reversed activated HSCs to quiescent cells, which suggested that naringin was indeed active component that rendered the GSG's effect to inhibit or reverse HSC activation. Naringin belongs to citrus flavinoids and was found to display strong anti‐inflammatory, antioxidant and antitumor activities 16, 17. Raha *et al*. found that naringin induces autophagy‐mediated growth inhibition by down‐regulating the PI3K/Akt/mTOR cascade *via* activation of MAPK pathways in AGS cancer cells 18. Since naringin was able to regulate mTOR‐autophagy pathway, we investigated their role in suppressive effect of naringin on HSC activation.

Autophagy plays an important role in HSCs activation. Along with HSCs activation, autophagy flux is up‐regulated. Autophagy may supply energy for activation of HSCs by delivering triglyceride and other components in lipid drops from autophagosomes to lysosomes for degradation 19, 20. Hernández‐Gea *et al*. found that inhibition of autophagic function in primary mouse HSCs and mice following reduced fibrogenesis and matrix accumulation, which suggested autophagy promoted HSCs activation 21. Similar to previous study 18, we found that naringin induced autophagic activity in HSCs. However, autophagic activation induced by narigin did not induce HSCs activation. On the contrary, HSCs activation was inhibited. It suggested HSCs deactivation was not because of autophagy but naringin. The deactivation of HSCs caused by naringin was further enhanced by the inhibition of autophagy, which indicated deactivation of HSCs caused by naringin was not because of the autophagic activation.

**Figure 1:**
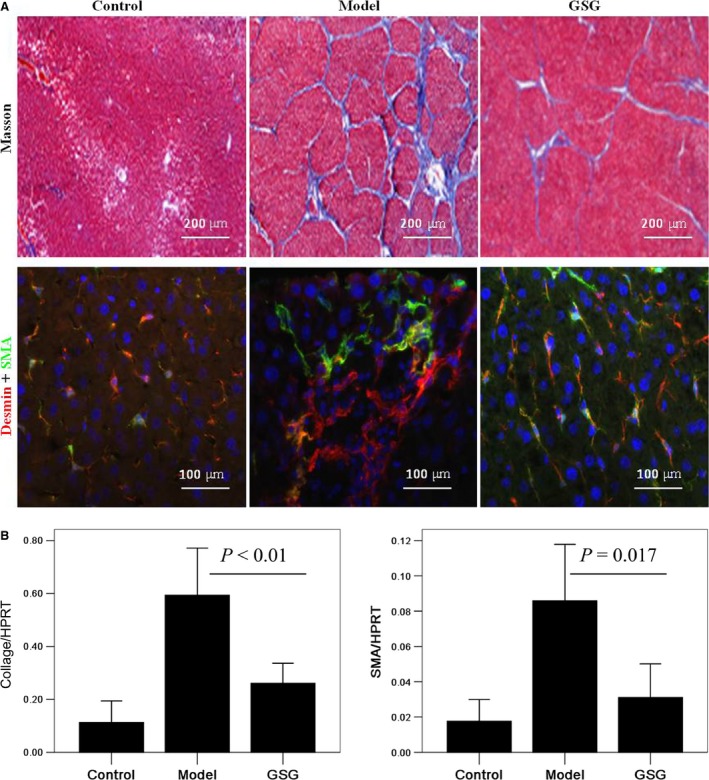
GSG depresses liver fibrosis and activation of hepatic stellate cells in mouse model. Control group (*n* = 10): mice were intraperitoneally injected with oil as the model group following fed with normal diet; Model group (*n* = 10): mice were intraperitoneally injected with a mixture of CCl_4_ (40%) and oil (60%) at a dose of 2 ml/kg body weight twice a week for 12 weeks following treated intragastrically with distilled water for 2 weeks besides normal diet; GSG group (*n* = 10): mice were treated as model group for 12 weeks following treated intragastrically with GSG at a dose of 4 g/(kg·day) for 2 weeks besides normal diet. **(A)** Liver fibrosis and cirrhosis in mice was detected by Masson staining. The expression of desmin and SMA in liver was detected by immunofluorescence. **(B)** The mRNA expression of collage‐I and SMA in liver were measured by quantitative polymerase chain reaction (qPCR). Data are presented from at least three independent experiments. GSG, Ganshuang granule; SMA, smooth muscle actin.

**Figure 2:**
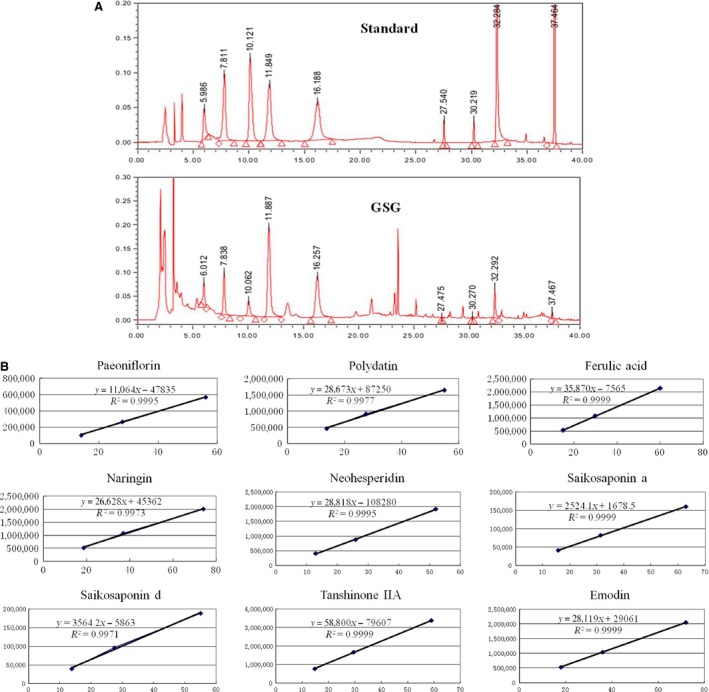
Identification of active component of GSG by HPLC. **(A)** In the HPLC fingerprint, the different components were eluted at different time‐points. **(B)** The standard curves between concentration and peak area were drawn and concentration of different components in GSG was calculated by peak area. Nine active components in GSG were identified such as paeoniflorin (peak 1, 2.224 mg/g), polydatin (peak 2, 1.557 mg/g), ferulic acid (peak 3, 0.733 mg/g), naringin (peak 4, 6.128 mg/g), neohesperidin (peak 5, 2.783 mg/g), saikosaponin a (peak 6, 0.039 mg/g), saikosaponin d (peak 7, 0.469 mg/g), tanshinone IIA (peak 8, 0.484 mg/g) and emodin (peak 9, 0.102 mg/g). Data are presented from at least three independent experiments. GSG, Ganshuang granule; HPLC, high performance liquid chromatography.

**Figure 3:**
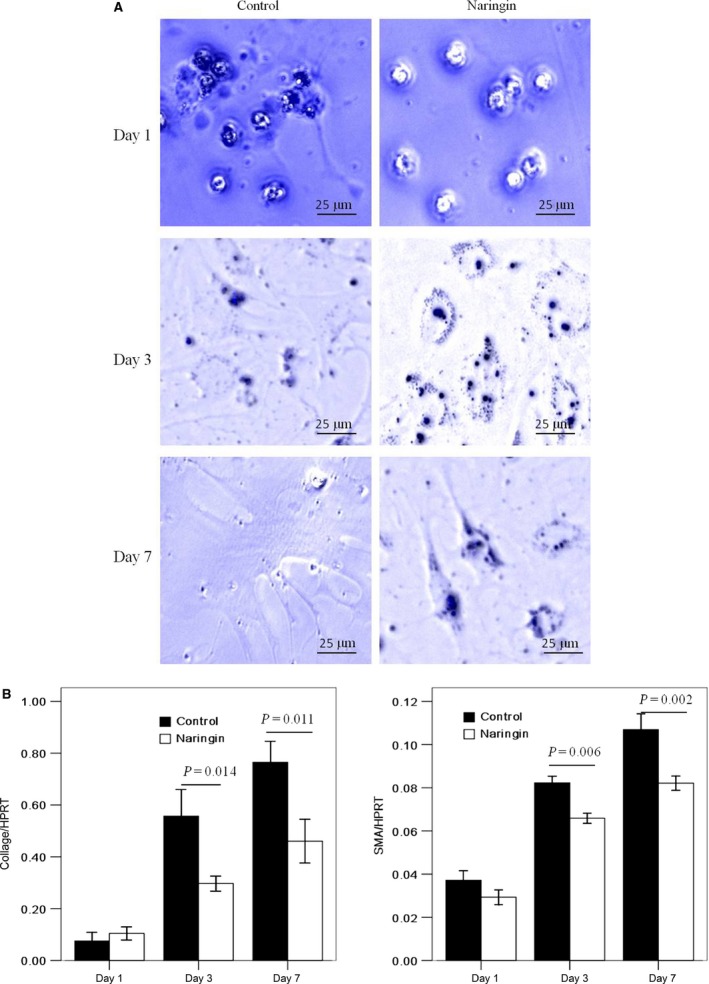
Naringin suppresses activation of primary mouse HSCs. Primary mouse HSCs were isolated and cultured on plastic dish for 1, 3, 7 days, respectively. Naringin (20 ng/ml) was added into day 1 HSCs for 24 hrs and day 3 activating or day 7 fully activated HSCs for 48 hrs. **(A)** Lipid drops and dendrite‐like processes in HSCs were observed in UV light of microscope at day 1, 3, 7 after isolation. A representative image was shown from three independent experiments. **(B)** The mRNA expression of collage‐I and SMA in HSCs were measured by qPCR. Data are presented from at least three independent experiments. HSC, hepatic stellate cell; SMA, smooth muscle actin.

**Figure 4:**
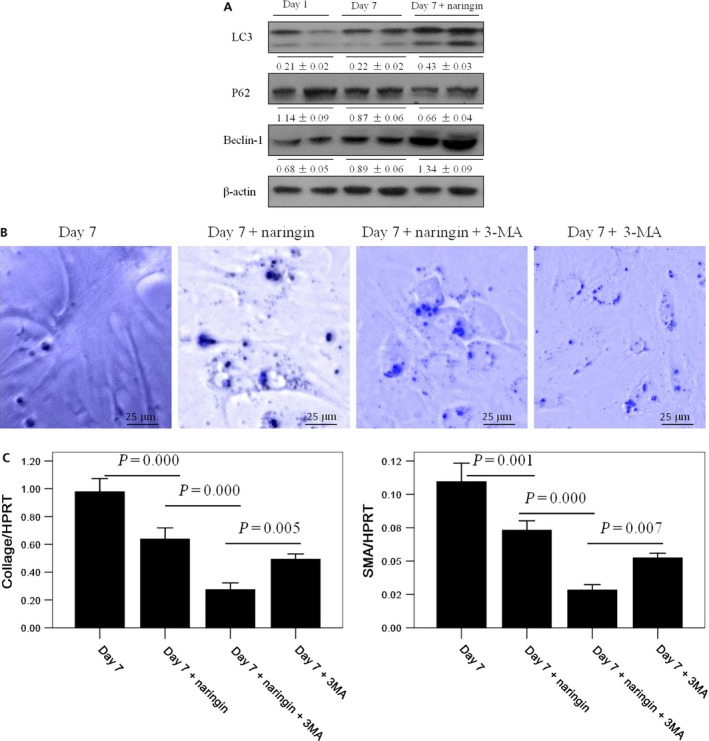
The suppressive effect of naringin on HSCs activation is further enhanced by inhibition of autophagy. Primary mouse HSCs were isolated and cultured for 1 or 7 days, respectively. Naringin (20 ng/ml) was added into day 7 fully activated HSCs for 48 hrs. 3‐methyladenine (3‐MA, 750 ng/ml) was added into day 7 fully activated HSCs for 2 hrs. **(A)** Protein expression levels of autophagy‐related proteins, including LC3, P62 and Beclin‐1were measured by western blotting in HSCs from different groups. Densitometry analysis was performed using ImageJ software, and the relative levels of protein were normalized to β‐actin. A representative blot for two samples from each group is shown. **(B)** Lipid drops and dendrite‐like processes in HSCs were observed in UV light of microscope in different groups. A representative image was shown from three independent experiments. **(C)** The mRNA expression of collage‐I and SMA in HSCs were measured by qPCR. Data are presented from at least three independent experiments. HSC, hepatic stellate cell. SMA, smooth muscle actin.

**Figure 5:**
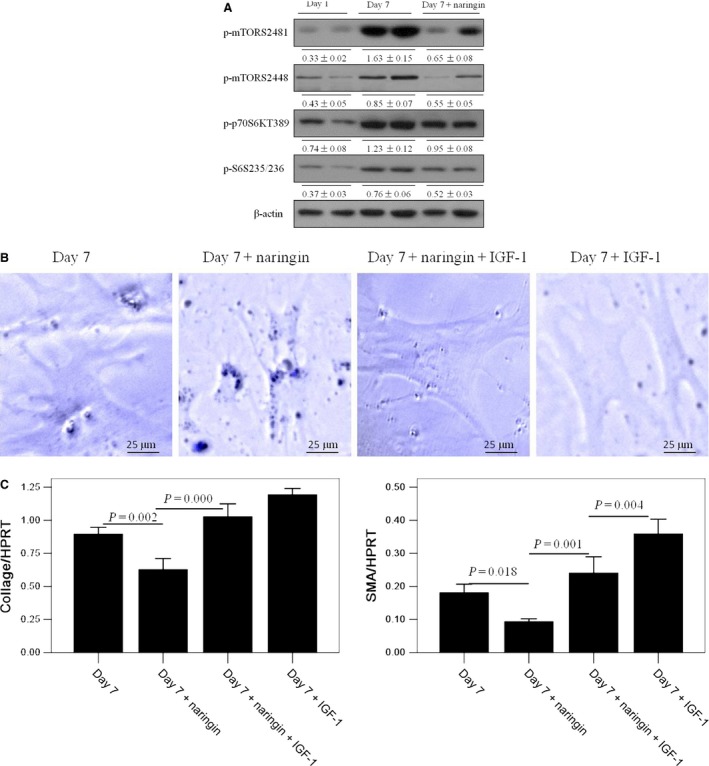
Naringin suppresses activation of HSCs through mTOR pathway. Primary mouse HSCs were isolated and cultured for 1 and 7 days, respectively. Naringin (20 ng/ml) was added into day 7 fully activated HSCs for 48 hrs. Insulin‐like growth factor‐1 (IGF‐1, 500 ng/ml) was added into day 7 fully activated HSCs for 2 hrs. **(A)** Protein expression levels of mTOR pathway, including mTOR, p70S6K and S6 were measured by western blotting in HSCs from different groups. Densitometry analysis was performed using ImageJ software, and the relative levels of protein were normalized to β‐actin. A representative blot for two samples from each group is shown. **(B)** Lipid drops and dendrite‐like processes in HSCs were observed in UV light of microscope in different groups. A representative image was shown from three independent experiments. **(C)** The mRNA expression of collage‐I and SMA in HSCs were measured by qPCR. Data are presented from at least three independent experiments. HSC, hepatic stellate cell; mTOR, mammalian target of rapamycin; SMA, smooth muscle actin.

**Figure 6:**
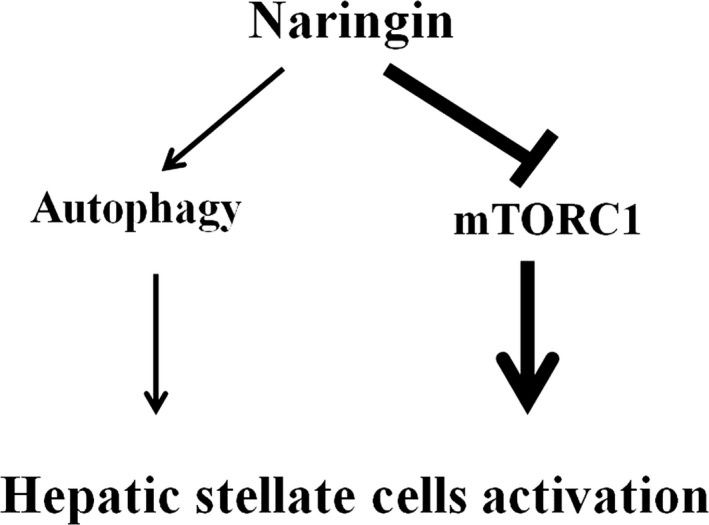
A model based on our study, illustrates that naringin suppresses activation of HSCs for anti‐fibrosis effect by inhibition of mTOR. Naringin has a dual role in HSCs activation: suppressing HSCs activation through inhibition of mTORC1 pathway, meanwhile promoting HSCs activation through activation of autophagy. However, the suppressive effect of naringin on mTORC1 overwhelmed the activated effect of naringin on autophagy. HSC, hepatic stellate cell; mTOR, mammalian target of rapamycin.

Activation of mTOR pathway is an important signalling pathway stimulating cell growth and proliferation. Growing researches show that mTOR pathway plays an important role in HSCs activation 22, 23, 24. Wang *et al*. found that inhibition of mTORC1 ameliorated fibrosis in rat model of bile duct ligation 23. Li *et al*. found that luteolin suppressed activation of hepatic stellate cells and liver fibrosis by targeting AKT/mTOR/p70S6K and TGFβ/Smad signalling pathways 24. We found that naringin down‐regulated mTOR pathways in activated HSCs. In addition, the suppressive effect of naringin on HSCs activation was significantly attenuated or completely abolished when mTOR pathway was activated, which suggested that naringin was able to suppress activation of HSCs for anti‐fibrosis effect *via* inhibition of mTOR pathway (Figs. 1, 2, 3, 4, 5, 6).

In summary, this study adds to the general understanding of anti‐fibrosis mechanism of GSG and provides new sights into the importance of naringin in regulating HSCs activation partially through mTOR pathway. Furthermore, this study gives a basis for clinical application of GSG. In the future, Chinese medicine will make a contribution to therapeutic strategy of liver fibrosis and cirrhosis.

## Conflicts of interest

The authors declare no conflict of interest.
